# The impact of the BreastScreen NSW transition from film to digital mammography, 2002–2016: a linked population health data analysis

**DOI:** 10.5694/mja2.52566

**Published:** 2025-01-12

**Authors:** Rachel Farber, Nehmat Houssami, Kevin McGeechan, Alexandra L Barratt, Katy JL Bell

**Affiliations:** ^1^ Sydney School of Public Health the University of Sydney Sydney NSW; ^2^ The Family Planning Australia Research Centre Sydney NSW

**Keywords:** Early detection of cancer, Mass screening, Mammography

## Abstract

**Objectives:**

To assess the impact of the transition from film to digital mammography in the Australian national breast cancer screening program.

**Study design:**

Retrospective linked population health data analysis (New South Wales Central Cancer Registry, BreastScreen NSW); interrupted time series analysis.

**Setting:**

New South Wales, 2002–2016.

**Participants:**

Women aged 40 years or older with breast cancer diagnosed during 2002–2017 who had been screened by BreastScreen NSW and for whom complete follow‐up information until the end of the recommended re‐screening interval was available.

**Intervention:**

Transition from film to digital mammography; 2009 defined as transition year (digital mammography becomes dominant screening modality).

**Main outcome measures:**

Population rates of screen‐detected cancer, interval cancer, recalls, and false positive findings.

**Results:**

The study cohort comprised 967 573 women; of the 2 741 555 screens, 1 535 184 used film mammography (2002–2010) and 1 206 371 used digital mammography (2006–2016). The screen‐detected cancer rate was 4.86 (95% confidence interval [CI], 4.75–4.97) cases per 1000 screens with film mammography and 6.11 (95% CI, 5.97–6.24) cases per 1000 screens with digital mammography (unadjusted difference, 1.24 [95% CI, 1.06–1.41] cases per 1000 screens). The interval cancer rate was 2.56 (95% CI, 2.48–2.64) cases per 1000 screens with film mammography and 2.84 (95% CI, 2.75–2.94) cases per 1000 screens with digital mammography (unadjusted difference, 0.27 [95% CI, 0.15–0.40] cases per 1000 screens). With the transition to digital mammography, the screen‐detected cancer rate increased by 0.07 per 1000 screens, the sum of the decline in the invasive cancer rate (–0.21 cases per 1000 screens) and the rise in the ductal carcinoma in situ detection rate (0.28 cases per 1000 screens); during 2009–2015, it increased by 0.18 cases per 1000 screens per year. With the transition to digital mammography, the interval cancer rate increased by 0.75 cases per 1000 screens (invasive cancer: by 0.69 cases per 1000 screens); during 2009–2015, it declined by 0.13 cases per 1000 screens per year. The recall rate increased by 8.02 per 1000 screens and the false positive rate by 7.16 per 1000 screens following the transition; both rates subsequently declined to pre‐transition levels.

**Conclusions:**

The increased screen‐detected cancer rate following the transition to digital mammography was not accompanied by a reduction in interval cancer detection rates.

**The known**: Breast cancer screening around the world has switched from film to digital mammography. The impacts of this change on health outcomes are unknown.

**The new**: In NSW, increased cancer detection with the new technology was predominantly of ductal carcinomas in situ; the detection of invasive cancer initially declined slightly. The interval cancer detection rate also increased, particularly the detection of invasive cancers. An initial increase in the rate of recalls for further assessment was largely attributable to the increased false positive result rate.

**The implications**: The transition from film to digital mammography may have increased the detection of indolent cancers.

Breast cancer screening programs moved from film to digital mammography during the first decade of the 21st century, primarily for workflow reasons,^1^, ^2^ but improved cancer detection was also anticipated.^3^ After the change, it was found that digital mammography indeed detected more cancers than film mammography,^4^, ^5^, ^6^, ^7^ particularly ductal carcinomas in situ (DCIS), with smaller increases in invasive cancer detection.^8^ However, most studies found no change in the reporting of interval cancers, suggesting that increased screening detection might predominantly be of slower growing (or even non‐progressive) cancers.^8^ At the same time, the transition to digital mammography was accompanied by a clear increase in recall rates, mostly related to false positive findings.^8^

The only analysis of the transition from film to digital mammography undertaken in Australia^9^ was not a population‐level study. BreastScreen Australia is a government‐funded screening program in which women aged 50−74 years are invited for screening every two years; women aged 40–49 years can also participate but are not formally invited. Two or more radiologists (or specifically trained breast physicians) independently read two‐view mammograms; the results are combined in a single recommendation about the need for further assessment to determine the presence of breast cancer.^10^

Overseas comparisons of digital and film mammography have used cancer rates without adjustment for time. However, as the two screening modalities were used during different time periods, rate differences could be confounded by time‐dependent factors, such as the changing background risk of breast cancer.^11^ In Australia, the transition from film to digital mammography in BreastScreen was largely undertaken during 2009 and 2010. As sufficient time has since elapsed, we assessed the impact of the transition of the Australian national screening program to digital mammography in an interrupted time series analysis adjusted for confounding by temporal trends.^12^

## Methods

The national breast screening program in Australia, BreastScreen Australia, commenced in 1988, and screening was nationally available by 1991.^10^ BreastScreen is nationally governed but, like most health care in Australia, it is implemented at the state level. A screening episode commences with the initial attendance for screening and includes any recalls for technical repeat screening or the assessment of abnormalities detected by screening mammography. A screening episode is completed when a recommendation is made to return the woman to routine screening or a cancer diagnosis is made. An invasive breast cancer or DCIS diagnosed after additional investigations is classified as a screen‐detected cancer. If cancer is not detected, it may be recommended that the woman return to two‐yearly, annual, or early screening (within six months). Cancers diagnosed after a recommendation to return to screening but before the end of the recommended screening interval are classified as interval cancers.^8^

### Study cohort and data sources

The study cohort has been described in detail elsewhere.^13^ Briefly, we used data linkage to construct a study cohort comprising two overlapping groups of women: all women screened by BreastScreen NSW from its inception (1 March 1988) to 31 December 2017; and all women aged 40 years or older (ie, screening‐eligible) diagnosed with DCIS or invasive breast cancer notified to the NSW Central Cancer Registry during 1 January 1988 – 31 December 2017. In 2024, New South Wales included an estimated 8.2 million people, or about one‐third of the Australian population.^14^

The BreastScreen NSW dataset comprises data for all program‐related mammography undertaken in NSW, including screening results, results of follow‐up investigations, and the final recommendations for each screening round. In order to achieve similar study time frames for film and digital mammography screening, we limited our study to women screened during 1 January 2002 – 31 December 2016; however, we used all BreastScreen NSW screening data since its inception to determine whether screening episodes for individual women during the study period were initial or subsequent screens.

BreastScreen NSW and NSW Central Cancer Registry data were probabilistically linked by the NSW Centre for Health Record Linkage (CHeReL).^15^ The registry uses pathology laboratory, hospital, radiotherapy and medical oncology departments, aged care facility, and Registry of Births, Deaths, and Marriages data to histologically verify all cases of breast cancer diagnosed in NSW residents, including DCIS. The linkage of the two datasets allowed us to determine the screening modality and subsequent outcomes for each woman with a high level of capture and accuracy.

Computed radiography screening was used only briefly by BreastScreen; it exposed women to higher radiation doses and provided lower quality images than digital mammography.^16^ We therefore excluded women screened using this modality from our analysis.

### Outcomes

Screen‐detected breast cancers were defined as those diagnosed on the basis of a positive screening result; we report the number of women diagnosed with screen‐detected breast cancer, including DCIS, per 1000 screens. Interval cancers were defined as cancers diagnosed after a negative screening mammography result and before the next scheduled screening, or cancers that were symptomatic on a subsequent screen; we report the number of interval cancers, including DCIS, per 1000 screened women. The recall rate was defined as the number of women with positive screening results recalled for further assessment per 1000 screened women, and the false positive rate as the difference between the recall and screen‐detected cancer rates.

### Statistical analysis

We initially included all screens undertaken by BreastScreen NSW during 2002–2016, regardless of whether the women had been invited to participate in screening. We stratified screens as initial or subsequent screening round screens, as the cancer detection rate differs by screening round.^17^ We stratified both screen‐detected and interval cancers as DCIS or invasive cancers, as the prognosis differs by cancer type.^18^, ^19^ We excluded screens for which the follow‐up time was not at least as long as the interval to the next recommended screen; that is, insufficient for ascertaining whether an interval cancer had been detected. Cancers diagnosed beyond the time point for the next recommended screening were deemed to be “cancers in lapsed screening participants”, in line with BreastScreen terminology. We report summary statistics that include cancers in lapsed screening participants, but did not include these cases in our regression analyses. We report overall rates of screen‐detected cancers, interval cancers, recalls, and false positive findings per 1000 screens for film and digital mammography, each with 95% confidence intervals (CIs). We calculated unadjusted rate differences in logistic regression models with robust standard errors.

We assessed rate changes in interrupted time series analyses, using 2009 as the transition time point, the main period of transition from film to digital mammography. An interrupted time series is a strong quasi‐experimental design for estimating changes attributable to an intervention (here: change in screening modality). We compared outcome rates after the introduction of digital imaging with those predicted by temporal trends preceding its introduction, which reflect changes unrelated to the screening modality, such as screening women at a younger age in more recent years.^12^, ^20^, ^21^, ^22^, ^23^ We used segmented autoregression to statistically estimate aggregate changes in annual rates between the film and digital mammography periods. The models estimate the baseline temporal trend during the film period, the immediate effects of the switch from film to digital mammography, and the temporal trend after the introduction of digital mammography. We used the segmented regression equation:

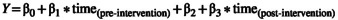

β_0_ is the estimated baseline rate with film mammography; β_1_ is the estimated annual temporal change in rate during the film mammography period (2002–2009); β_2_ is the estimated change in rate attributed to the change to digital mammography; and β_3_ is the estimated additional change in rate during the digital mammography period (2009–2015). We report β_0_, β_1_, β_2_, and β_3_, overall and by screen type (initial or subsequent screens); we depict rates by time derived from independent models for initial and subsequent screens as graphs.^20^, ^24^ We performed the linked data analyses in SAS 9.4, the interrupted time series analysis in SAS Studio 3.1 (PROC AUTOREG).

### Ethics approval

The NSW Population and Health Service Research Ethics Committee approved the study (HREC 2019/ETH08688). Access to the datasets used was approved by their respective data custodians.

## Results

During 2002–2017, BreastScreen NSW undertook a total of 5 200 928 screens; we excluded 920 903 computed radiography screens, 78 408 screens for which the modality was unknown, and 1 460 062 otherwise eligible screens for which interval cancer follow‐up was incomplete. Our study cohort comprised 967 573 women aged 40 years or older with breast cancers diagnosed during 2002–2017 who had been screened by BreastScreen NSW and for whom complete follow‐up information until the end of the recommended re‐screening interval was available (Box 1). Of the 2 741 555 screens included in our analysis, 1 535 184 used film mammography (2002–2010) and 1 206 371 used digital mammography (2006–2016) (Box 2). A total of 30 120 breast cancers were diagnosed in these women: 14 834 screen‐detected cancers and 7366 interval cancers, as well as 7920 lapsed screen participant cancers (Supporting Information, table 1).

Box 1 **Study cohort derivation: women aged 40 years or older who were diagnosed with breast cancer (ductal carcinoma in situ or invasive) notified to the NSW Central Cancer Registry and screened by BreastScreen NSW during 1 January 1988 – 31 December 2017**

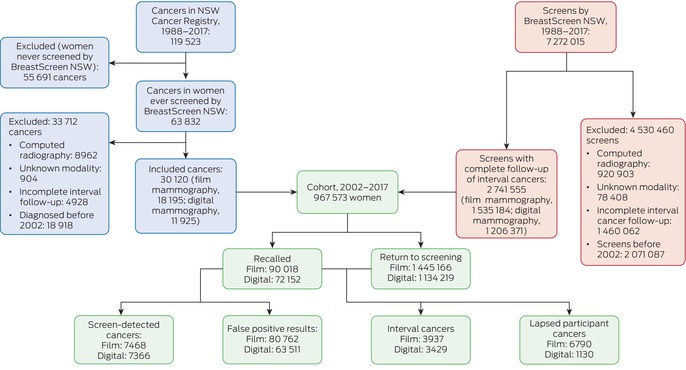

Box 2 BreastScreen NSW screening (initial and subsequent screens) using film or digital mammography, 2002–2016, by calendar year*

**Table:** 

Year	Film mammography	Digital mammography	Film and digital mammography
2002	286 853	0	286 853
2003	282 419	0	282 419
2004	263 450	0	263 450
2005	165 178	0	165 178
2006	172 003 (95.7%)	7687 (4.3%)	179 690
2007	159 785 (95.4%)	7792 (4.7%)	167 577
2008	135 815 (84.4%)	25 163 (15.6%)	160 978
2009	50 172 (38.6%)	79 904 (61.4%)	130 076
2010	19 509 (15.8%)	104 167 (84.2%)	123 676
2011	0	122 897	122 897
2012	0	160 584	160 584
2013	0	202 267	202 267
2014	0	220 073	220 073
2015	0	274 632	274 632
2016*	0	1205	1205
Total	1 535 184	1 206 371	2 741 555

* Number of screens during 2016 with sufficient follow‐up time for interval cancer detection.

### Screen‐detected cancers

The screen‐detected cancer rate was 4.86 (95% CI, 4.75–4.97) cases per 1000 screens with film mammography (2002–2010) and 6.11 (95% CI, 5.97–6.24) cases per 1000 screens with digital mammography (2006–2016), an unadjusted difference of 1.24 (95% CI, 1.06–1.41) cases per 1000 screens. The invasive cancer rate increased by 0.70 (95% CI, 0.55–0.86) cases per 1000 screens, the DCIS rate by 0.53 (95% CI, 0.45–0.60) per 1000 screens (Box 3). In the time series model adjusted for temporal trends, the initial background rate was 4.65 per 1000 screens; it rose by 0.05 cases per 1000 screens per year during 2002–2009 (Box 4).

Box 3 **BreastScreen NSW screening outcomes, by modality (digital, 2006–2016; film mammography, 2002–2010) and screen type (initial or subsequent): unadjusted logistic regression analysis**

**Table:** 

	Digital mammography		Film mammography		Difference
Outcome	Number	Rate per 1000 screens (95% CI)	Number	Rate per 1000 screens (95% CI)	Rate per 1000 screens (95% CI)
**Screens**	1 206 371	—	1 535 184	—	—
Initial screen	167 339	—	218 492	—	—
Subsequent screen	1 039 032	—	1 316 692	—	—
**Screen‐detected cancer**	7366	6.11 (5.97–6.24)	7468	4.86 (4.75–4.97)	1.24 (1.06 to 1.41)
Initial screen	1496	8.94 (8.49–9.39)	1421	6.50 (6.17–6.84)	2.43 (1.87 to 2.99)
Subsequent screen	5870	5.65 (5.51–5.79)	6047	4.59 (4.48–4.71)	1.05 (0.87 to 1.24)
Ductal carcinoma in situ	1581	1.31 (1.24–1.37)	1196	0.77 (0.73–0.82)	0.53 (0.45 to 0.60)
Initial screen	315	1.88 (1.67–2.09)	227	1.03 (0.90–1.17)	0.84 (0.59 to 1.09)
Subsequent screen	1266	1.21 (1.15–1.28)	969	0.73 (0.68–0.78)	0.48 (0.40 to 0.56)
Invasive	5785	4.79 (4.67–4.91)	6272	4.08 (3.98–4.18)	0.70 (0.55 to 0.86)
Initial screen	1181	7.05 (6.65–7.45)	1194	5.46 (5.15–5.77)	1.59 (1.08 to 2.09)
Subsequent screen	4604	4.43 (4.30–4.55)	5078	3.85 (3.75–3.96)	0.57 (0.40 to 0.74)
Interval cancer	3429	2.84 (2.75–2.94)	3937	2.56 (2.48–2.64)	0.27 (0.15 to 0.40)
Initial screen	392	2.34 (2.11–2.57)	504	2.31 (2.11–2.51)	0.03 (–0.27 to 0.34)
Subsequent screen	3037	2.92 (2.82–3.03)	3433	2.61 (2.52–2.69)	0.31 (0.18 to 0.45)
Ductal carcinoma in situ	345	0.28 (0.25–0.31)	291	0.18 (0.16–0.21)	0.09 (0.05 to 0.13)
Initial screen	42	0.25 (0.17–0.32)	30	0.13 (0.08–0.18)	0.11 (0.02 to 0.20)
Subsequent screen	303	0.29 (0.25–0.32)	261	0.19 (0.17–0.22)	0.09 (0.05 to 0.13)
Invasive cancer	3084	2.55 (2.46–2.64)	3646	2.37 (2.29–2.45)	0.18 (0.06 to 0.29)
Initial screen	350	2.09 (1.87–2.31)	474	2.16 (1.97–2.36)	–0.07 (–0.37 to 0.21)
Subsequent screen	2734	2.63 (2.53–2.72)	3172	2.40 (2.32–2.49)	0.22 (0.09 to 0.35)
**Recalls**	72 152	59.8 (59.4–60.2)	90 018	58.6 (58.3–59.0)	1.17 (0.60 to 1.73)
Initial screen	21 709	129.7 (128.1–131.3)	22 358	102.3 (101.1–103.6)	27.4 (25.4 to 29.4)
Subsequent screen	50 443	48.6 (48.1–49.0)	67 660	51.4 (51.0–51.8)	–2.83 (–3.39 to –2.27)
**False positive findings**	63 511	52.6 (52.2–53.0)	80 762	52.6 (52.2–53.0)	0.03 (–0.49 to 0.57)
Initial screen	20 089	120.0 (118.5–121.6)	20 663	94.6 (93.3–95.8)	25.5 (23.5 to 27.5)
Subsequent screen	43 422	41.8 (41.4–42.2)	60 099	45.6 (45.3–46.0)	–3.85 (–4.37 to –3.32)

CI = 95% confidence interval.

Box 4 BreastScreen NSW screening outcomes, 2002–2015, by period and screen type (initial or subsequent): interrupted time series analysis*

**Table:** 

Outcome	Baseline rate (β_0_), per 1000 screens	Change in rate, 2002–2009 (β_1_), per 1000 screens/year	Immediate change in rate (β_2_), per 1000 screens	Change in rate, 2009–2015 (β_3_), per 1000 screens/year
**Screen‐detected cancers**	4.65	0.05	0.07	0.18
Initial screen	5.61	0.24	0.28	0.06
Subsequent screen	4.48	0.02	0.04	0.19
Ductal carcinoma in situ	0.67	0.03	0.28	0.01
Initial screen	0.94	0.02	0.08	0.14
Subsequent screen	0.62	0.03	0.30	–0.01
Invasive cancers	3.98	0.02	–0.21	0.16
Initial screen	4.67	0.22	0.20	–0.08
Subsequent screen	0.62	0.03	0.30	–0.01
**Interval cancers**	2.52	0.01	0.75	–0.13
Initial screen	1.89	0.11	–0.25	–0.13
Subsequent screen	2.63	–0.01	0.90	–0.13
Ductal carcinoma in situ	0.17	0.01	0.08	0.00
Initial screen	0.11	0.01	0.11	–0.01
Subsequent screen	0.18	0.01	0.09	–0.01
Invasive	2.35	0.01	0.69	–0.13
Initial screen	1.78	0.11	–0.26	–0.14
Subsequent screen	1.88	0.08	0.02	–0.16
**Recalls**	55.6	0.78	8.02	–2.97
Initial screen	83.7	5.25	31.0	–14.2
Subsequent screen	49.8	0.47	17.1	–6.34
**False positive results**	49.8	0.73	7.16	–2.95
Initial screen	79.0	4.19	1.59	–2.35
Subsequent screen	44.7	0.24	8.27	–3.15

* Cancer detection rates by time derived from independent models for initial and subsequent screens by cancer type (screen‐detected or interval cancer) are provided in Box 5, and for screen‐detected invasive cancer and ductal carcinomas in situ in Box 6. Recall and false positive rates by time derived from independent models for initial and subsequent screens are provided in Box 7.

With the transition to digital mammography, the screen‐detected cancer rate increased by 0.07 cases per 1000 screens, the sum of the decline in the invasive cancer rate (–0.21 per 1000 screens) and the rise in the DCIS detection rate (0.28 per 1000 screens). During 2009–2015, the screen‐detected cancer rate increased by 0.18 cases per 1000 screens per year, comprising rises of 0.16 invasive cancers and 0.01 DCIS per 1000 screens per year (Box 4).

For initial screens, the screen‐detected cancer rate increased by 0.28 cases per 1000 screens followed by an increase of 0.06 cases per 1000 screens per year. For subsequent screens, the screen‐detected cancer rate increased by 0.04 cases per 1000 screens followed by an increase of 0.19 cases per 1000 screens per year (Box 4, Box 5).

Box 5 BreastScreen NSW screening outcomes, 2002–2015, by cancer type (screen‐detected or interval cancer) and screen type (initial or subsequent)*

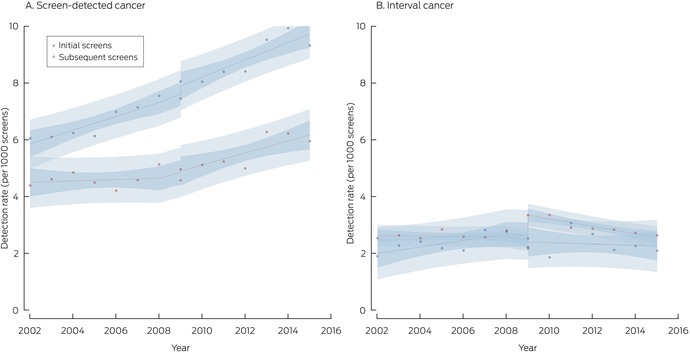

* Points indicate actual values, with fitted curves as dashed lines; dark blue region indicates 95% confidence interval for predicted trend, light blue regions 95% confidence interval for predicted values. Data for 2016 were not included, as the numbers of screens during 2016 with sufficient follow‐up time for interval cancer detection were small.

### Interval cancers

The interval cancer rate was 2.56 (95% CI, 2.48–2.64) cases per 1000 screens with film mammography (2002–2010) and 2.84 (95% CI, 2.75–2.94) cases per 1000 screens with digital mammography (2006–2016), an unadjusted difference of 0.27 (95% CI, 0.15–0.40) cases per 1000 screens. The invasive cancer rate increased by 0.18 (95% CI, 0.06–0.29) cases per 1000 screens, the DCIS rate by 0.09 per 1000 screens (95% CI, 0.05–0.13) (Box 3). In the time series model adjusted for temporal trends, the initial background rate was 2.52 cases per 1000 screens; it rose by 0.01 cases per 1000 screens per year during 2002–2009 (Box 4).

With the transition to digital mammography, the interval cancer rate increased by 0.75 cases per 1000 screens, comprising rises in the invasive cancer (0.69 per 1000 screens) and DCIS detection rates (0.08 per 1000 screens). During 2009–2015, the interval cancer detection rate declined by 0.13 cases per 1000 screens per year, entirely attributable to the decline in the interval invasive cancer detection rate (Box 4).

### Recalls and false positive results

The recall rate was 58.6 (95% CI, 58.3–59.0) per 1000 screens with film mammography and 59.8 (95% CI, 59.4–60.2) per 1000 screens with digital mammography, an unadjusted difference of 1.17 (95% CI, 0.60–1.73) cases per 1000 screens. The recall rate for initial screens increased from 102 (95% CI, 101–104) to 130 (95% CI, 128–131) per 1000 screens, an unadjusted difference of 27.4 (95% CI, 25.4–29.4) recalls per 1000 screens. The recall rate for subsequent screens declined from 51.4 (95% CI, 51.0–51.8) to 48.6 (95% CI, 48.1–49.0) per 1000 screens, an unadjusted difference of –2.83 (95% CI, –3.39 to –2.27) recalls per 1000 screens (Box 3).

Most recalls were related to false positive findings, the overall rate of which did not change (both modalities: 52.6 per 1000 screens; unadjusted difference between digital and film mammography, 0.03 [95% CI, –0.49 to 0.57] false positive findings per 1000 screens). The false positive finding rate for initial screens increased from 94.6 (95% CI, 93.3–95.8) to 120 (95% CI, 118–122) per 1000 screens (unadjusted difference, 25.5 [95% CI, 23.5–27.5] per 1000 screens); the rate for subsequent screens declined from 45.6 (95% CI, 45.3–46.0) to 41.8 (95% CI, 41.4–42.2) per 1000 screens (unadjusted difference, –3.85 [95% CI, –4.37 to –3.32] per 1000 screens (Box 3).

In the interrupted time series adjusted for temporal trends, the initial background recall rate was 55.6 per 1000 screens; it rose by 0.78 cases per 1000 screens per year during 2002–2009. With the transition to digital mammography, the recall rate increased by 8.02 per 1000 screens during 2009–2015, it declined by 2.97 cases per 1000 screens per year (initial screens, –14.2 cases per 1000 screens per year; subsequent screens, –6.34 cases per 1000 screens per year) (Box 4, Box 7).

The temporal changes in false positives were similar to those seen in recall rates with an immediate increase of 7.16 per 1,000 screens, and a yearly decrease of 2.95 per 1000 screens with digital mammography. However, the pattern was more pronounced in subsequent screens than initial screens. (Box 4, Box 7).

Box 6 BreastScreen NSW screen‐detected cancers, 2002–2015, by cancer type (ductal carcinoma in situ or invasive cancer) and screen type (initial or subsequent)*

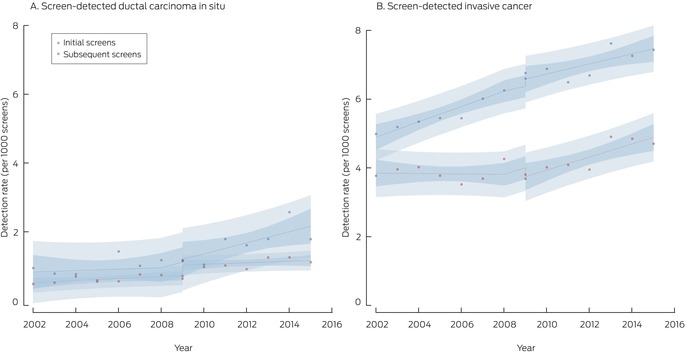

* Points indicate actual values, with fitted curves as dashed lines; dark blue region indicates 95% confidence interval for predicted trend, light blue regions 95% confidence interval for predicted values. Data for 2016 were not included, as the numbers of screens during 2016 with sufficient follow‐up time for interval cancer detection were small.

Box 7 BreastScreen NSW recall and false positive finding rates, 2002–2015, by screen type (initial or subsequent)*

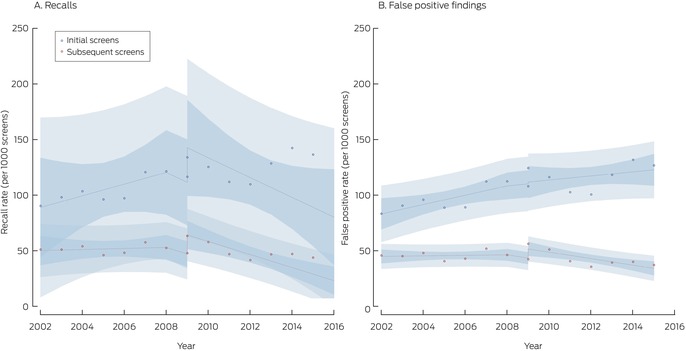

* Points indicate actual values, with fitted curves as dashed lines; dark blue region indicates 95% confidence interval for predicted trend, light blue regions 95% confidence interval for predicted values. Data for 2016 were not included, as the numbers of screens during 2016 with sufficient follow‐up time for interval cancer detection were small.

### Age and screening round

For all screens, the difference between film and digital mammography screen‐detected cancer rates declined by ten‐year age group from 40–49 to 60–69 years, but rose again for women aged 70 years or older. The difference for interval cancer detection was smaller for women aged 50–69 years than for those aged 40–49 years, but was highest for women aged 70 years or older. Recall rates declined with increasing age (Supporting Information, table 2).

## Discussion

The BreastScreen NSW transition from film to digital mammography during 2006–2010 was accompanied by an increase in the screen‐detected cancer rate, especially during initial screens; the increase was primarily attributable to the higher DCIS detection rate. Our interrupted time series analysis, adjusted for secular changes in cancer diagnosis rates, indicated that the screen‐detected invasive cancer rate declined at the transition point before rising again. If most of the additional screen‐detected cancers were clinically important, interval cancer rates would be expected to decline, but they initially increased, particularly for subsequent screens. Some additionally detected cancers might otherwise have been detected at subsequent screenings, or they might have been detected later as interval cancers, especially invasive cancers. It is also possible that some cancers would not ever have been detected during life, particularly low grade DCIS, which may indicate overdiagnosis since the transition. The reason for the increase in the interval cancer detection rate at the transition is unclear, but the initial decline in the screen‐detected invasive cancer rate could be one factor.

Digital mammography provides better image quality and reduces the radiation exposure of screened women compared with film‐based screening, and also improved workflow efficiency.^25^, ^26^ However, the high contrast and resolution of digital mammography not only accentuates lesions, but also normal tissue architecture. Although lesions may be easier to distinguish, reading and interpreting digital mammograms requires training.^27^ The increased recall rate immediately after the transition to digital mammography in NSW may indicate that radiologists saw lesions and tissue structures that had been less visible in film mammography. With continuous changes in screening technology, and the need for a period of learning after each change, recall rates may not stabilise if new technologies continue to be introduced.^28^ The increase in the recall rate was especially marked for women aged 40–49 years. Recall and false positive finding rates are generally higher in this age group than for older women, partly because of denser breast tissue in younger women.^8^ We found that the increases in the screen‐detected cancer rate was greatest for women aged 40–49 years or 70 years or older, while the increase in interval cancer detection rate was greatest for women aged 70 years or older.

The initially increased recall rate after the modality transition exceeded Australian breast screening standards — fewer than 10% of women aged 50–69 years attending their first screening episode are recalled for assessment^10^ — but declined with time. Nevertheless, the increase could have had a substantial impact on the wellbeing of women who were recalled.^29^, ^30^ Increased recall rates after the transition to digital mammography in other countries also declined after an adjustment period.^31^, ^32^ The crude screen‐detected cancer rates for NSW (digital mammography, 6.11 per 1000 screens; film mammography, 4.86 per 1000 screens) were in the middle of the range of values reported overseas, while the crude interval cancer detection rates (digital mammography, 2.84 per 1000 screens; film mammography, 2.56 per 1000 screens) corresponded to the high end of overseas estimates;^8^ the reason for this difference requires further investigation. We have described the tumour characteristics of screen‐detected and interval cancers in our cohort elsewhere.^33^

We report the first interrupted time series analysis of the impact on breast screening program outcomes of the transition from film to digital mammography. This approach facilitates assessment of the effectiveness of population‐level health interventions after adjusting for time‐dependent confounders.^11^ Confounders that change relatively slowly over time, such as population age distribution and obesity, are taken into account by the long term temporal trend estimate.^34^ We could separately estimate changes in screen‐detected and interval cancer rates attributable to secular population changes and to the transition in screening modality. Our study is also the first to compare film and digital mammography outcomes at the population level in Australia, apart from one case–control study that could not estimate population rates.^35^ Our analysis of linked statewide data for histologically verified outcomes facilitated a robust analysis.

### Limitations

We excluded from our analysis a substantial number of computed radiography screens, screens for which the modality was unknown, and otherwise eligible screens for which interval cancer follow‐up was incomplete. These exclusions reduced the number of included screens undertaken during 2005–2012, and also meant that the proportion of included screens of women aged 40–49 years was smaller for digital than film screens (Supporting Information, table 2). As age influenced screening outcomes by modality, we may have underestimated increases in recall and screen‐detected cancer rates associated with the transition to digital mammography. Our analysis of administrative data was limited by the data collected and recorded; it would be desirable to stratify screening outcomes by mammographic density, but it is not assessed by BreastScreen NSW. Confounders that changed rapidly at the same time as the transition in screening technology may not have been accounted for in our analysis. Nonetheless, population‐based health care data sources provide opportunities for relatively rapid and cost‐efficient quasi‐experimental evaluation and outcomes research. Finally, screen‐detected and interval cancer rates have not been robustly validated as surrogate outcome measures of breast cancer mortality.^36^

### Conclusion

Both screen‐detected cancer and interval cancer rates increased with the move by BreastScreen NSW from film to digital mammography. Adjusting for the background rates reduced the increase in the screen‐detected cancer rate but not that of interval cancer detection. The health benefits of the screening modality transition may have been smaller than anticipated, and were accompanied by increased recall and false positive finding rates, and possibly by overdiagnosis. The effects of future changes in mammography technology, including the introduction of breast tomosynthesis, should be rigorously evaluated.

## Open access

Open access publishing facilitated by The University of Sydney, as part of the Wiley ‐ The University of Sydney agreement via the Council of Australian University Librarians.

## Competing interests

No relevant disclosures.

## Data sharing

Access to the data and analysis files underlying this report is permitted only with the explicit permission of the approving human research ethics committees and the data custodians. Analysis of linked data is currently authorised at only one location.

## Supporting information


Supplementary results
