# Supplementary material for: The impact of the BreastScreen NSW transition from film to digital mammography, 2002–2016: a linked population health data analysis
Source: Med J Aust. 2025 Jan 12;222(2):82–90. doi: 10.5694/mja2.52566 (PMC11787811; doi:10.5694/mja2.52566)
Supplement: Supplementary file 1 — Supplementary results [file MJA2-222-82-s001.pdf]

Supporting Information

Supplementary results

This appendix was part of the submitted manuscript and has been peer reviewed. It is posted as supplied by the authors.

Appendix to: Farber R, Houssami N, McGeechan K, et al. The impact of the BreastScreen NSW transition from film to digital mammography, 2002–2016: a linked population health data analysis. *Med J Aust* 2025; doi: 10.5694/mja2.52566.

Table 1. Summary statistics for lapsed-screen cancers

A lapsed screen is when the next screen is undertaken after the recommended return to screen date (or there is no subsequent screen). There was a slightly higher rate of lapse screens with film 1,016,287 (66.2%) than with digital 721,622 (59.8%). However, it is inappropriate to compare lapsed cancers between film and digital mammography because by definition the cancer has occurred beyond the screening interval. i.e., because film has been around for longer there is a longer time for a cancer to develop since the lapsed screen. Below is a table of the time of the diagnosis of the lapsed cancer since the recommended return to screening date. While this gives a more accurate comparison for shorter periods from recommended screening, there is increased bias for longer periods from recommended screening since film has been around for longer. The comparison for 1 year from recommended screening is least biased, and the comparison for >5 years is most biased.

	**Digital**			**Film**		
	Number	Rate per 1,000 screens	Rate per 1000 lapsed screens	No.	Rate per 1000 screens	Rate per 1000 lapsed screens
**Screens**	1,206,371			1,535,184		
**Lapsed screens**	721,622 (59.8%)	598.1		1,016,287 (66.2%)	662.0	
**Lapsed cancers**	1,130	0.94	1.57	6,790	4.42	6.68
**Years lapsed**						
1 year	467	0.39	0.65	924	0.60	0.91
2 years	244	0.20	0.34	667	0.43	0.66
3 years	159	0.13	0.22	675	0.44	0.66
4 years	129	0.11	0.18	659	0.43	0.65
5 years	42	0.03	0.06	611	0.40	0.60
> 5 years	26	0.02	0.04	2603	1.70	2.56

Table 2. BreastScreen NSW screening outcomes, by modality (digital, 2006–2016; film mammography, 2002–2010) and screen type (initial or subsequent) and age group: unadjusted logistic regression analysis

	All screens					Initial screens					Subsequent screens				
	Digital mammography		Film mammography		Difference	Digital mammography		Film mammography		Difference	Digital mammography		Film mammography		Difference
Outcome/age group (years)	Number	Rate*	Number	Rate*	Rate*	Number	Rate*	Number	Rate*	Rate*	Number	Rate*	Number	Rate*	Rate*
**Screens**															
40–49	88 385	—	204 695	—	—	55 776	—	86 539	—	—	32 609	—	118 156	—	—
50–59	500 944	—	637 177	—	—	89 127	—	95 547	—	—	411 817	—	541 630	—	—
60–69	481 653	—	491 883	—	—	18 956	—	27 510	—	—	462 697	—	464 373	—	—
70 or older	135 389	—	201 427	—	—	3480	—	8894	—	—	131 909	—	192 533	—	—
**Screen-detected cancers**															
40–49	447	5.06	522	2.55	2.51	340	6.10	290	3.35	2.74	107	3.28	232	1.96	1.32
50–59	2477	4.94	2683	4.21	0.73	811	9.10	676	7.08	2.02	1666	4.05	2007	3.71	0.34
60–69	3171	6.58	2839	5.77	0.81	282	14.9	306	11.1	3.75	2889	6.24	2533	5.45	0.79
70 or older	1271	9.39	1424	7.07	2.32	63	18.1	149	16.8	1.35	1208	9.16	1275	6.62	2.54
Ductal carcinoma in situ															
40–49	129	1.46	105	0.51	0.95	86	1.54	48	0.55	0.99	43	1.32	57	0.48	0.84
50–59	599	1.20	452	0.71	0.49	177	1.99	114	1.19	0.79	422	1.02	338	0.62	0.40
60–69	634	1.32	440	0.89	0.42	38	2.00	46	1.67	0.33	596	1.29	394	0.85	0.44
70 or older	219	1.62	199	0.99	0.63	14	4.02	19	2.14	1.89	205	1.55	180	0.93	0.62
Invasive cancers															
40–49	318	3.60	417	2.04	1.56	254	4.55	242	2.80	1.76	64	1.96	175	1.48	0.48
50–59	1878	3.75	2231	3.50	0.25	634	7.11	562	5.88	1.23	1244	3.02	1669	3.08	–0.06
60–69	2537	5.27	2399	4.88	0.39	244	12.9	260	9.45	3.42	2293	4.96	2139	4.61	0.35
70 or older	1052	7.77	1225	6.08	1.69	49	14.1	130	14.6	–0.54	1003	7.60	1095	5.69	1.92

	All screens					Initial screens					Subsequent screens				
	Digital mammography		Film mammography		Difference	Digital mammography		Film mammography		Difference	Digital mammography		Film mammography		Difference
**Interval cancers**															
40–49	287	3.25	529	2.58	0.66	161	2.89	198	2.29	0.60	126	3.86	331	2.80	1.06
50–59	1221	2.44	1516	2.38	0.06	163	1.83	210	2.20	–0.37	1058	2.57	1306	2.41	0.16
60–69	1 358	2.82	1316	2.68	0.14	50	2.64	68	2.47	0.17	1308	2.83	1248	2.69	0.14
70 or older	563	4.16	576	2.86	1.30	18	5.17	28	3.15	2.02	545	4.13	548	2.85	1.29
Ductal carcinoma in situ															
40–49	38	0.43	39	0.19	0.24	19	0.34	13	0.15	0.19	19	0.58	26	0.22	0.36
50–59	131	0.26	107	0.17	0.09	17	0.19	12	0.13	0.07	114	0.28	95	0.18	0.10
60–69	125	0.26	94	0.19	0.07	5	0.26	5	0.18	0.08	120	0.26	89	0.19	0.07
70 or older	51	0.38	51	0.25	0.12			—	—	—	—	—	—	–	–
Invasive cancers															
40–49	249	2.82	490	2.39	0.42	142	2.55	185	2.14	0.41	107	3.28	305	2.58	0.70
50–59	1090	2.18	1 409	2.21	0.04	146	1.64	198	2.07	–0.43	944	2.29	1211	2.24	0.06
60–69	1233	2.56	1 222	2.48	0.08	45	2.37	63	2.29	0.08	1188	2.57	1159	2.50	0.07
70 or older	512	3.78	525	2.61	1.18	17	4.89	28	3.15	1.74	495	3.75	497	2.58	1.17
**Recalls**															
40–49	9556	108	15 704	76.7	31.4	7213	129	8 414	97.2	32.1	2343	71.8	7290	61.7	10.2
50–59	32 286	64.4	38 541	60.5	3.96	11 895	133	10 383	109	24.8	20 391	49.5	28 158	52.0	–2.47
60–69	23 252	48.3	25 602	52.0	3.77	2220	117	2678	97.4	19.8	21 032	45.5	22 924	49.4	–3.91
70 or older	7058	52.1	10 171	50.5	1.64	381	109	883	99.3	10.2	6677	50.6	9288	48.2	2.38

* Per 1000 screens.
